#  Are there geographic and socio-economic differences in incidence, burden and prevention of malaria? A study in southeast Nigeria

**DOI:** 10.1186/1475-9276-8-45

**Published:** 2009-12-23

**Authors:** Obinna Onwujekwe, Benjamin Uzochukwu, Nkem Dike, Chijioke Okoli, Soludo Eze, Ogoamaka Chukwuogo

**Affiliations:** 1 Department of Health Administration and Management, College of Medicine, University of Nigeria, Enugu, Nigeria; 2 Health Policy Research Group, Department of Pharmacology and Therapeutics, College of Medicine, University of Nigeria, Enugu, Nigeria; 3 Department of Community Medicine, College of Medicine, University of Nigeria, Enugu, Nigeria; 4 Roberta Buffett Center for International & Comparative Studies, Northwestern University, Chicago, USA

## Abstract

**Rationale:**

It is not clearly evident whether malaria affects the poor more although it has been argued that the poor bear a very high burden of the disease. This study explored the socioeconomic and geographic differences in incidence and burden of malaria as well as ownership of mosquito nets.

**Methods:**

Structured questionnaires were used to collect information from 1657 respondents from rural and urban communities in southeast Nigeria on: incidence of malaria, number of days lost to malaria; actions to treat malaria and household ownership of insecticide treated and untreated mosquito nets. Data was compared across socio-economic status (SES) quartiles and between urban and rural dwellers.

**Results:**

There was statistically significant urban-rural difference in malaria occurrence with malaria occurring more amongst urban dwellers. There was more reported occurrence of malaria amongst children and other adult household members in better-off SES groups compared to worse-off SES groups, but not amongst respondents. The average number of days that people delayed before seeking treatment was two days, and both adults and children were ill with malaria for about six days. Better-off SES quartile and urban dwellers owned more mosquito nets (p < 0.05) (treated and untreated).

**Conclusion:**

Malaria occurs more amongst better-off SES groups and urban dwellers in southeast Nigeria. Deployment of malaria control interventions should ensure universal access since targeting the poor and other supposedly vulnerable groups may exclude people that really require malaria control services.

## Introduction

Malaria has frequently been linked with poverty and reducing the burden of malaria is increasingly becoming a global priority [1]. Malaria is one of the leading causes of mortality and morbidity in Nigeria and particularly affects children under 5 years [2]. Almost 3% of disability adjusted life years are due to malaria mortality globally, 10% in Africa [3]. The economic burden of malaria illness on households accounts for almost 50% of total economic burden of illnesses in malaria holo-endemic communities [4-7]. Also, living in malaria-endemic regions places an economic burden on households even if they do not actually suffer an episode of malaria and reducing malaria improves households' living standards [8]. However, it has been noted that poor people bear a disproportionate burden of the disease and have poor health seeking habits [9,3,12].

The evidence about the differential occurrence of malaria amongst different socio-economic status groups is mixed and it is not clear why malaria should affect certain SES groups especially the poor more than others. Poor people are purported to be at increased risk both of becoming infected with malaria and of becoming infected more frequently [13]. Although there are strong *a priori *reasons for believing that the burden of malaria is greatest on the poor, the evidence in the literature to support this is mixed and often contradictory [10,6,14,8,3]. Previous studies reported that incidence of malaria is typically lower at the very top of the wealth distribution, but the relationship is not strong after controlling for confounding factors [1]. Also, it was reported that some studies found no clear difference in fever incidence based on wealth status, but did show significant disparities in both the consequences of malaria and in the use of malaria prevention and treatment services [14]. People from poor SES groups had more parasitaemia than people from better-off SES groups but using self-reported malaria or fever as the measure of malaria infection, there was a lack of association with SES [15]. However, it was reported that 58% of the cases occur in the poorest 20% of the world's population and these patients receive the worst care and have catastrophic consequences from their illness [3]. Results from a previous study in Nigeria suggest heavier malaria burden on the poor than on the rich because individuals with a mean income of below N300/day (<US$1/day) were less likely to perceive malaria as a preventable disease, more likely to report having fever presently, and suffered significantly more bouts of malaria per month when compared with individuals earning greater than N300 per day [16]. Other studies found a substantially higher prevalence of malaria infection among the poorest population groups [17,18] and that the poorest were most susceptible to contracting malaria [19,20].

It has also been argued that rural dwellers have higher risk factors and greater malaria burden compared to urban dwellers [21]. Between 6% and 28% of the malaria burden may occur in cities, which comprise less than 2% of the African surface [3]. It has been found that the incidence of malaria is lower in urban than rural settings [22]. Anaemia and parasitaemia both showed that rural dwellers have highest risk factors for malaria in children under five years of age, even after controlling for bed net use [21]. Residents of urban areas have potentially protective variables against malaria risk such as education, income, good environment, etc. [23].

There could also be a relationship between prevention of malaria and its incidence because members of the poor socio-economic groups live in dwellings that offer little protection against mosquitoes and are less able to afford insecticide-treated nets [13]. Hence, it is argued that better-off SES groups and urban dwellers usually possess more malaria preventive tools, thus they should have less incidence of malaria. On the other hand, because of both physical and financial barriers, the poor and rural dwellers may not own and use adequate quantities of malaria preventive tools such as mosquito nets. Furthermore, they are less likely to be able to pay either for effective malaria treatment or for transportation to a health facility capable of treating the disease [13]. Use of mosquito nets was found to be negatively and significantly associated with parasitaemia[15].

The paper examines the socio-economic and geographic differences in incidence, length of malaria morbidity, and ownership of mosquito nets for the prevention of malaria. The paper provides new information that will help in a better appreciation of inequities in burden and control of malaria amongst different population groups and highlight areas that require interventions to decrease the burden to all population groups.

## Materials and methods

### Study area

The study was conducted in Anambra State in Southeast Nigeria. The communities selected for the household survey were Awka and Onitsha (urban), and Enugwu-ukwu and Okpoko (rural). The study sites were selected with the help of policy makers in the Anambra State Ministry of Health (MOH) as being representative of rural and urban areas in the state. Unpublished MOH reports show that the transmission rates in both the urban and rural areas are similar and occur all year round there. However the study was conducted during the rainy season when transmission rate is highest. These factors enabled the fair comparison of data from urban and rural areas for exploration of geographic inequities. Each site area had a full complement of providers from hospitals to itinerant drug providers and herbalists. The rural areas like the urban areas have electricity and other social amenities such as tarred roads and schools. Awka is the State capital and Onitsha is the commercial capital of the state.

### Study design and sampling

This study was a cross-sectional community household survey. The listing of the households in each of the study areas was used to produce a sampling frame. The software for population survey in EPI Info 6 was used for sample size calculation. The parameters that were used for sample size calculation were a power of 80%, 95% confidence level. The calculations assumed that all the socio-economic groups used the services equally. The last parameter was the study population, which was the number of people with malaria in the study sites. Anambra MOH estimates an average of 6% monthly malaria incidence rate in the state. Hence, using the minimum projected population of each rural site at 30,000 people and each urban area at 60,000 people, it was estimated that a minimum of 1800 and 3600 people would have malaria monthly in each rural and urban area. The estimated minimum sample sizes were 400 per urban site and 350 per rural site, but a uniform sample size of 420 was chosen for both urban and rural areas in order to take care of refusals and incomplete questionnaires.

### Data collection

Pre-tested interviewer administered questionnaires were used to elicit information from the randomly selected households. In each selected household the primary healthcare giver usually a female (the wife) or in her absence the male head of the household was interviewed using the questionnaire. Data was collected on socio-economic and demographic data of the respondents; incidence of self-reported malaria among respondents, other adults (other adult household residents apart from the respondent), and children in the selected households one month preceding the survey; duration of the illnesses (and ability to perform normal functions); and ownership of different types of mosquito nets. Children in the context of this paper refer to every child below 13 years of age.

### Data analysis

The data was pooled across the two urban areas and two rural areas respectively, so as to yield urban and rural data sets, which were then subjected to statistical analysis. Cross-tabulations, testing of means and non-parametric tests were used to compare the key variables across different socio-economic and geographic groups. An asset-based socio-economic status (SES) index developed using principal components analysis was used to examine whether there were systematic SES differences in the variables [20]. The variables that were included in the index were household ownership of functional radio, fridge, TV, bicycle, motorcycle and motor car as well as weekly per capita household food value. Comparison of urban with rural data sets was used to examine for geographic differences in the variables.

## Results

A total of 1657 questionnaires out of the 1680 administered were complete, analyzed and used for presentation of results. In both the rural and urban areas, majority of the respondents were the primary female healthcare giver (wives) (Table 1). The average age of the respondents was 40 years and most of them had formal education. The major occupation of the respondents was petty trading. Most of the respondents owned a television, radio and fridge.

**Table 1:** Socio-economic and demographic characteristics of respondents and their households

	Rural N = 863	Urban N = 794
**Information on respondent**		
No of household residents: Mean (SD)	4.9 (2.3)	5.4 (2.2)
Sex: Females n %	739 (85.6)	669 (84.3)
Mean Age of respondent (SD)	39.5 (15.4)	39.8 (14.0)
Attended school: n %	732 (84.8)	706 (88.9)
Years of education: Mean (SD)	8.6 (4.7)	9.3 (5.5)

*Status in households n %		
**Female head of household	95 (11.0)	56 (7.1)
Male head of household	101(11.7)	87 (10.9)
***Wife	521 (60.4)	527 (66.4)
Grandmother	21 (2.4)	33 (4.2)
Representative	125 (14.5)	91 (11.5)

**Occupation of household head **n %		
Farmer	73 (8.5)	14 (1.8)
Petty trading	285 (33.0)	260 (32.8)
Government worker	51 (5.9)	131 (16.5)
Employed in private sector	41 (4.8)	48 (6.0)
Medium/big business	48 (5.6)	57 (7.2)
Self-employed professional	99 (11.5)	104 (13.0)
Unemployed	222 (25.4)	157 (19.8)
Others	44 (5.1)	23 (2.9)

**Household Items Owned **n %		
Radio	793 (91.9)	728 (91.7)
Fridge	489 (56.7)	559 (70.4)
TV	678 (78.6)	690 (86.9)
Bicycle	31 (3.6)	76 (9.6)
Motorcycle	60 (7.0)	77 (9.7)
Motorcar	135 (15.6)	200 (25.2)

*Status in household refers to the position held in that household.

**One who is the breadwinner of the family whose huband is late, away or one who is divorced.

***Woman catered for by her husband.

### Rural-Urban and SES differences in occurrence of malaria

More adults compared with children had self-reported malaria one month prior to the survey (Table 2). It was found that 38.5% and 35% of the urban and rural respondents and 9.2% and 14.4% of other adults belonging to the households had self-reported malaria respectively one month to the date of the interview (Table 2). There was no statistically significant difference in malaria occurrence in urban and rural areas amongst the respondents (p > 0.05), but there was amongst other adult household members and amongst children. There was no statistically significant SES difference in malaria occurrence amongst the respondents. However, reported malaria incidence increased as SES increased in case of children and other adult household members.

**Table 2:** SES and urban-rural differences in the incidence of malaria among adults and children

	Respondents had malaria in past month n (%)	Other adults had malaria (%)	Whether a child had malaria n (%)
**Urban-rural differences**			

Rural n = 863	332 (38.5)	79 (9.2)	124 (14.4)
Urban n = 794	278 (35.0)	114 (14.4)	144 (18.1)
Equity ratio (R:U)	1.1	0.6	0.8
X^2 ^(p-value)	2.02 (.16)	14.7 (.005)	10.76 (.029)

**SES differences**			

SES quartiles			
Q1 n = 408 most poor	144 (35.3)	37 (9.1)	59 (14.5)
Q2 n = 407 very poor	142 (34.9)	43 (10.6)	58 (14.3)
Q3 n = 407 poor	155 (38.0)	49 (12.0)	67 (16.5)
Q4 n = 407 least poor	155 (38.0)	61 (15.0)	79 (19.4)
Equity ratio (Q1:Q4)	0.9	0.6	0.7
X^2 ^(p-value)	1.51 (.68)	14.93 (.002)	13.1 (.01)

More than 97% of all adults and children sought treatment for malaria. There was no urban-rural as well as SES difference in whether or not to seek treatment for malaria for both adults and children. The average number of days delayed before seeking treatment which was higher in amongst the rural dwellers compared to the urban dwellers (p < 0.05) but was not statistically significantly different across SES quartiles (Table 3). The number of days that people were ill when they could not perform their normal functions was slightly higher in the urban area when compared to rural area, but the difference was not statistically significant. The most poor SES were sick for greater number of days compared to the least poor but the differences across SES groups was not statistically significant (Table 3).

**Table 3:** Inequities in number of days that adults and children were ill with malaria

	No of days adults (respondents and 'other' adults) delayed before seeking treatment Mean (SD)	No of days adults(respondents and 'other' adults) ill with malaria Mean (SD)	No of days children ill with malaria Mean (SD)	No of days children delayed before treatment was sought Mean (SD)
**Urban-rural differences**				

Rural	2.3 (1.6)	6.4 (4.7)	5.5 (2.5)	2.3 (2.9)
Urban	2.0 (1.3)	6.6 (4.5)	6.1 (3.6)	1.8 (1.2)
Equity ratio (R:U)	1.1	1.0	0.9	1.3
X^2 ^(p-value)	6.2 (.013)	.23 (6.3)	1.0 (.32)	.03 (.87)

**SES differences**				

SES quartiles				
Q1 most poor	2.1 (1.4)	7.4 (6.4)	6.1 (2.7)	2.3 (2.9)
Q2 very poor	2.2 (1.3)	6.4 (3.3)	5.3 (2.4)	1.9 (1.2)
Q3 poor	2.0 (1.3)	6.1 (3.6)	6.1 (4.0)	2.3 (2.7)
Q4 least poor	2.3 (1.6)	6.3 (4.6)	5.8 (3.0)	1.8 (1.5)
Equity ratio (Q1:Q4)	0.9	1.2	1.1	1.3
X^2 ^(p-value)	2.5 (.48)	6.2 (.10)	2.6 (.46)	1.7 (.64)

Table 4 shows that urban dwellers owned more of all types of nets compared to rural dwellers. However, while there was generally high level of ownership of untreated window and door nets, the converse was true for treated and untreated bed nets. Also, ownership of nets increased with SES, although with slight aberration by Q3 for untreated door nets and Q2 for untreated and treated bed nets.

**Table 4:** Inequities in household ownership of mosquito nets

	Untreated window nets n (%)	Untreated door nets n (%)	Untreated bed-nets n (%)	Insecticide treated bets nets (ITNs) n (%)
**Urban-rural differences**				

Rural	211(24.4)	156(18.1)	27(3.1)	6(0.7)
Urban	334(42.1)	233(29.3)	68(8.6)	14(1.8)
Equity ratio	0.6	0.7	0.4	0.4
X^2 ^(p-value)	61.65 (.0001)	31.3 (.0001)	23.6 (.0001)	4.09 (.043)

**SES differences**				

SES quartiles				
Q1 most poor	57 (10.7)	43 (11.3)	13 (13.8)	3 (15.0)
Q2 very poor	106 (19.9)	76 (19.9)	27 (28.7)	6 (30.0)
Q3 poor	179 (35.6)	134 (35.2)	24 (23.5)	3 (15.0)
Q4 least poor	191 (35.8)	128 (35.6)	30(31.9)	8 (40.0)
Equity ratio	0.3	0.3	0.4	0.4
X^2 ^(p-value)	134.2 (.0001)	79.1 (.0001)	7.4 (0.60)	3.6 (.31)

## Discussion

The findings from this paper showed that there were SES differences in occurrence of self-reported malaria with more incidences amongst better-off SES groups. More adults (minus respondents) from better-off SES groups had more incidence of malaria. This differed from other studies which showed a higher prevalence of malaria among the poorest population groups [17,18]. From analysis of demographic and health surveys collected from 22 countries, the incidence of fever and its treatment were related to poverty in Sub Saharan Africa (SSA), with incidence typically lower at the very top of the wealth distribution[1]. Another study also found a statistical decline in malaria risk with increasing SES [20]. However, there are studies that did not find any association between self-reported malaria and SES but rather found a negative relationship between rate of parasitaemia and SES [15]. It is possible that illness perception is higher amongst better-off SES groups, while the poor are less likely to identify some symptoms of malaria as illness. Hence, there is increased or enhanced recognition and reporting of malaria by the richer SES. If better-off individuals were to report ill health more frequently than poorer individuals, any associations between SES and true malaria prevalence would be difficult to determine based on self-report alone, as higher (lower) SES individuals would over (under) report infection [15]. The results differ from others where it was found that incidence is typically lower with increasing SES, but the relationship may not be strong after controlling for confounding factors [1].

The findings showing that more urban dwellers compared to rural dwellers had self-reported malaria supports the assertion that "between 6% and 28% of the malaria burden may occur in cities, which comprise less than 2% of the African population"[3]. Urban areas also have more slums, where the living conditions could be worse than what is found in the rural areas. Hence, the finding from the study is not surprising since the urban study areas comprised some unclean areas and huge slums with high prevalence of mosquito breeding sites. Also, this is not unconnected to the environmental difference which is largely related to the building pattern common in many urban areas where there are limited good drainage systems to allow easy flow of water. Hence, in most cases, there is increase of risk factors in malaria infection in the urban areas compared to the rural areas.

Unlike previous studies that found that wealthier households were more likely to seek care or advice for malaria [1], the results showed that all SES groups as well as people living in urban and rural areas equally sought treatment when they had self reported malaria. However, one may query the quality of treatment they sought as previous studies found that higher SES groups were more likely to seek treatment from providers with better quality of services compared to poorer SES groups [1,6,8]. A study found that 10% and 29% of households reported at least one member being hospitalized due to malaria in one month, and 40% in Mozambique and 95% in South Africa had sought care before requiring hospitalization [20].

The number of days that people were ill, especially adults when they could not perform their normal functions had a direct consequence on reducing household production and increasing the indirect costs of the disease as part of the burden of malaria. With all residents of the two geographic areas and all SES groups losing the same number of days, the decreased production and cost of illness have a more profound effect on the poor and rural dwellers, most of whom depend on subsistence employment (and must in fact work daily) for them to be able to provide for basic household needs. The dire effect on the rural dwellers is magnified by the higher levels of delay before seeking care. The indirect costs of malaria are likely to be a key determinant of the disease's overall costs because adults give up activities to care for children when they are afflicted or the disease strikes the economically active population[6]. It has been found that in sub-Saharan Africa (SSA), sick adults loose 1-5 days per malaria episode [6,5,4]. Despite the findings that there was no SES difference in incidence and length of morbidity, there is a large body of evidence that payments for healthcare and other economic consequences of illness impose far greater burden on poor families than on high-income households [24]. The regressive nature of payments for malaria treatment has also been reported [25]. In Mozambique and South Africa, it has been found that malaria episodes lasted 4.4 and 7 days and there was labour substitution in 24% of cases in Mozambique [20]. The time sacrificed from caregivers of children from their own activities ranged from 1.1 to 2.7 days [20]. The social vulnerability in malaria burden requires better understanding for improving deployment, access, quality, and use of effective interventions [3].

The inequities in ownership of nets found in this study could be linked with the SES inequities in incidence but are contradictory to urban-rural differences in malaria incidence. It has been argued that the poor are less capable of protecting themselves from malaria [8], hence predisposing them to higher incidence and burden of malaria. Supporting the argument is the finding in this study that household acquisition and use of bed-nets among the different SES groups was found to be different with higher SES having more door, window, and bed nets. This is not unusual as the most poor have less financial capabilities to acquire malaria preventable tools such as bed or other nets. The urban-rural difference in the ownership of mosquito nets in favour of the urban dwellers could be because urban areas have more access to the malaria preventive tools such as bed nets due to the lopsided distribution channels for such products in the health system. The higher levels of ownership of nets by the better-off SES apparently was not associated with lower malaria incidence. However, one may query whether the nets were in good shape and/or properly used.

It is acknowledged that it is mostly self-reported malaria that is described in the paper as not all fevers are malaria. Laboratory diagnosis using either microscopy or Rapid Diagnostic Tests (RDTs) can improve the accuracy of diagnosis and help to understand differences of burden of actual malaria across different geographic and SES groups. The difference in respondents' incidences from that of other adults within their households is most likely a problem of recall about all adult malaria incidences within their households. It would have better results if the interview process had included a strategy for directly asking all adult household members whether they had malaria one month to the date of the interview. Apart from the likely problem of recall, another limitation was that in asking for the incidence of adult malaria, the questionnaire explored incidences amongst respondents and other adults separately. However in asking for the number of days that people were ill with malaria, the questionnaire did not differentiate between respondents and other adults in the households.

A possible limitation of comparing SES based on asset-index between different geographic groups from different data sets for urban and rural areas is the possible differential ownership of some of the assets in the rural and urban areas, unless concerted efforts are made to use assets that are owned equally in the two geographic areas. In this study the assets used were biased towards the urban area A previous study showed that an asset-based index is an effective alternative to consumption in determining the socio-economic gradient in malaria prevalence but recommended that further studies need to be undertaken on the relationship between parasitaemia, self-report, and fever [15]. It has also been suggested that longitudinal community-based studies to monitor the burden of malaria should be undertaken [22]. Hence, further studies that adopt better design than have been previously used are required to more accurately measure the incidence and burden of malaria across different SES and geographic groups. Also, future studies can explore inequities in incidence and morbidity of vulnerable groups such as pregnant women and children under-five instead of focusing on the general population. Such future studies should also examine burden of malaria more deeply by assessing the current status of not only on incidence but the cost of being ill (the productivity loss, absenteeism from school and other factors).

## Conclusion

There were mixed SES group and geographic differences in incidence of self reported malaria in this study, with the better-off SES groups in general reporting more malaria than worse-off SES groups, and with more malaria reported in urban areas compared to the rural areas. However, the presence of geographic and socio-economic inequities in the incidence and burden of malaria in the urban and rural areas can be remedied. Policy makers and programme managers should develop distribution channels for malaria prevention and treatment which can better protect everyone from malaria. This is supportive of the notion of universal access to malaria control interventions since targeting the poor and other supposedly vulnerable groups may actually exclude people that really require malaria control services and expose them to increased burden of the disease and potentially catastrophic costs that could lead to impoverishment or further impoverishment. The bottom-line is that for the overall equitable control of malaria, so as to remarkably decrease the burden of the disease, there should be mass deployment of malaria control intervention tools such as Artemisinin-based combination therapy (ACTs) and insecticide treated nets [8], without preference to any SES group or people residing in different geographic locations.

## Competing interests

The authors declare that they have no competing interests.

## Authors' contributions

OO conceived the study, participated in the design and performed statistical analysis. BU participated in the design of the study and coordination. ND participated in literature review and data collection. CO participated in the survey and literature review. SE participated in the survey. OC drafted the manuscript. All authors read and approved the final manuscript
